# Cell growth monitoring in a tetrapolar electrode configuration

**DOI:** 10.2478/joeb-2024-0009

**Published:** 2024-07-03

**Authors:** Jagbir Singh, Niranjan D. Khambete

**Affiliations:** 1 Biotechnology, IIT Madras, Chennai, India; 2 SCTIMST, Trivandrum, India

**Keywords:** Cell Growth Monitoring, bioimpedance, electrode, electrode Impedance Spectroscopy

## Abstract

There are various methods for cell growth monitoring. However, most of these methods have drawbacks, such as being invasive, not providing real-time results, or being costly. In this study, we present an alternate method of cell growth monitoring, which is low-cost, non-invasive, real-time, and uses Electrical Impedance Spectro-scopy (EIS). In this work, commercially available culture plates were fitted with custom tetrapolar electrodes, and mouse cells were cultured on them. The variation of culture media impedance, resulting from cell growth, proliferation and other metabolic activities, was recorded over a period of seven days. The results demonstrated an initial increase in impedance corresponding with the cell growth phase, followed by a decrease during the cell death (apoptosis) phase, as confirmed by microscope images. Overall, the results show that our method to monitor cell growth using tetrapolar electrodes is promising and can be further refined for related applications.

## Introduction

Cell growth monitoring is the prime requirement for all modern cell culture laboratories. It is vital for a biologist because the status of the monitoring is a firm indicative of the next course of action and a distinctive marker for the success or failure of intended usage. Other usage of cell growth monitoring extends to wound healing, migration, attachment and spreading, toxicology studies.

Various techniques exist for determining cell growth and viability for biological tissues, including microscopic counting, electronic particle counting, image analysis, in situ biomass monitoring, and dielectrophoretic cytometry [1]. These techniques are most effective when applied to a fixed volume sample from a suspension culture. Manual microscopic counting, although labor-intensive, offers the advantage of assessing cell viability when a suitable dye is employed. Electronic particle counting provides a rapid total cell count for replicate samples, but there may be data distortion if the sample contains significant cell debris or aggregates. Image analysis, facilitated by digital camera images obtained through microscopy, has seen significant advancement with the emergence of several commercially available software packages, replacing manual counting and viability determination [1]. Impedance methods have been explored in the past to monitor bacterial activity [2].

The typical EIS involves administering a predetermined voltage or current to the material and subsequently monitoring the resulting current or voltage. The stimulus can be applied as a step function, noise signal or a sinusoidal signal [3]. The work described is using the sinusoidal stimulus approach. A multi-frequency current is applied to the material and the resultant voltage is measured. The material being examined is consistently presumed to possess properties that do not change over time. Additionally, it is assumed that the material behaves linearly under electrical influence, enabling the application of the reciprocity theorem, allowing the positions where current is injected, to be interchangeably swapped with voltage measuring positions, without altering the voltage-to-current ratio [4]. This phenomenon holds significance, especially when employing small electrodes with high impedance, as non-linearity may manifest in the electrodes utilized for current injection and voltage measurement.

Tissues demonstrate characteristics of both conductors and dielectrics, containing both free and bound (fixed) charges. Consequently, tissue impedance encompasses both conductive and dielectric elements. The conductivity component represents the mobility of free charges, while the relative permittivity component elucidates the displacement of bound charges within the dielectric under an applied electrical field. Both relative permittivity and conductivity are observed to vary with frequency [5].

The biological tissues can be considered as a composite volume conductor comprising several spatially distributed tissues with different electrical properties. If a sinusoidal voltage is applied between the faces of a unit cubical tissue, the resulting conduction currents and displacement currents will be flowing. Conduction current is related to the ionic content and ionic mobility of the tissue. Displacement currents will contribute to time-varying electrical behavior of a tissue. Within the frequency range covered by this work, the cellular structure of tissue produces the dispersion [6]. At higher frequencies dispersion arises from the ability of molecules to re-orient in an applied field. Dispersions also occur at lower frequencies, but their origin is less clear. Cell membranes exhibit resistive and capacitive behavior, the frequency of measurement determines which one of these is dominant [7], [8].

One of the aims of this project was to develop an electrode configuration for cell growth monitoring. In this work, a four-electrode configuration has been proposed to inject 17 μA current through a pair of electrodes and measure voltage via another pair of electrodes. The recorded voltage is a representative of transfer impedance and plotted against time to monitor the impedance values [9], [10]. The excitation current value was empirically chosen to provide minimal necessary excitation, as well a possible extension to human applications, where excitation current determines patient safety [11], [12].

## Materials and methods

A 35 × 10 mm polystyrene culture plate (CELLSTAR^®^) was used for culturing cells and measurements. The plastic surface of CELLSTAR^®^ was treated, making it hydrophilic resulting in superior cell adhesion of cells and protein binding. L929 cell lines from ATCC^®^ were used for all experiments. The NCTC clone 929 (Connective tissue, mouse), clone of strain L was derived in March 1948. The parent L strain was derived from normal subcutaneous areolar and adipose tissue of a 100-day-old male C3H/An mouse. The strain tested negative for ectromelia virus (mouse pox).

The cell culture media used for culturing the L929 cells is Minimum Essential Medium (MEM) AutoMod^®^, modified for autoclaving. It contains amino acids salts (calcium chloride, potassium chloride, magnesium sulfate, sodium chloride, and monosodium phosphate) and glucose vitamins (folic acid, nicotinamide, riboflavin, B12). The MEM incorporates modifications to meet nutritional supplements of mammalian fibroblasts, including higher concentrations of amino acids so the medium closely matches the protein composition of a cultured mammalian cells. MEM has been used for cultivation of a wide variety of cells grown in monolayer and has been further modified by elimination of calcium to permit growth of cells in suspension culture[13].

**Fig.1:**
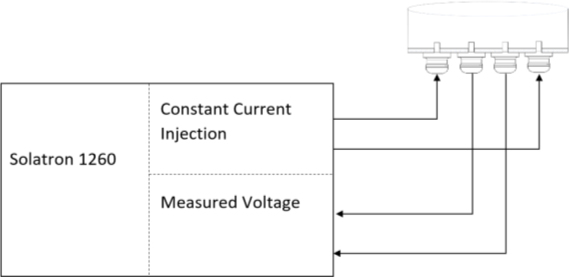
Equipment set up to measure and plot transfer impedance.

A Solatron^®^ 1260^®^ was used as Frequency Response Analyzer for measuring and plotting the impedance values with accuracy of 0.1%, 0.1°. Tissue impedance is measured using a set of metal electrodes. Four sets of electrodes were used in the tetrapolar impedance measurement method, Figure 1, where a constant current is injected through one pair of electrodes and the impedance-dependent voltage is measured with a second pair of electrodes.

**Fig.2:**
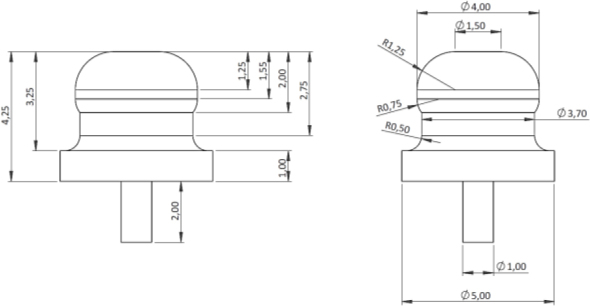
Drawing of the metal peg-based electrode used in the study. All electrode dimensions are in mm.

Electrodes used for all experiments in this study were made of medical grade stainless steel (316 L). The first electrode prototype consisted of four 316L wires, stretched in a row on top of the culture plate. The wire dimensions were 1 mm diameter, 30 cm length, spaced 5 mm apart, and placed symmetric to the culture plate center. However, this wire design had drawbacks, like stray capacitances and problem in connecting reliably to the measuring instrument, due the flexibility of the wire. Subsequently, a pin shaped electrode was developed, to address these issues, Figure 2.

**Fig.3:**
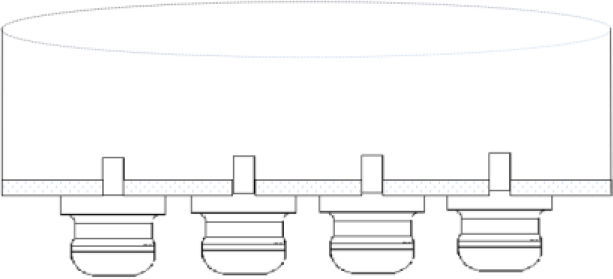
Cross-section representation of four electrodes fitted at the bottom of the dish.

For fitting the pin electrodes, holes were drilled in the culture plate and the electrodes were fitted at the bottom, Figure 3.

**Fig.4:**
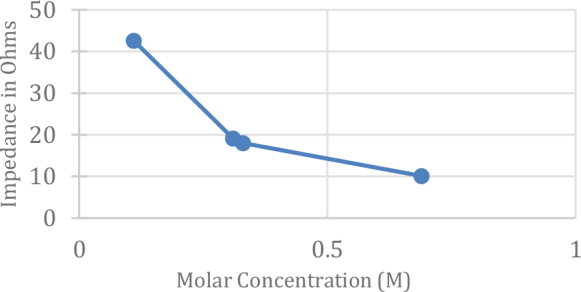
Impedance of the control (NaCl solution) at different Concentration at 1 kHz.

A biocompatible adhesive, GY 250 (a universal purpose unmodified medium viscous Bipenol A epoxy resin) cured with HY 2963 hardener (a Cycloaliphatic Amine) in the ratio of 2:1 was used to attach the electrode to the culture plates. NaCl solutions of varying concentration were used to get an idea of measurement robustness using this electrode set up. NaCl solutions of known concentration were put in the pin type electrode fitted dish and impedance values were recorded for various molar concentrations, Figure 4.

**Fig.5:**
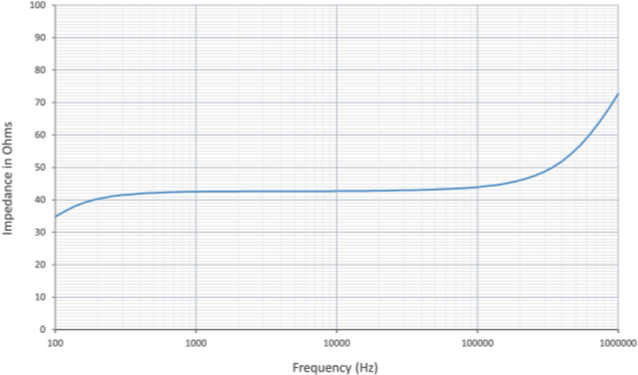
Variation of Impedance with frequency of 0.119 M NaCl solution.

Afterwards, an impedance scan through a frequency range of 100 Hz to 1 MHz was performed, Figure 5. Non-linearity in measured impedance were observed at frequencies below 500 Hz (due to polarization) and above 50 kHz due to stray inductance/capacitance of the wires making the measurement unreliable. As a result, the frequencies measurement and reported for this work are in between 1 kHz and 50 kHz.

**Fig.6:**
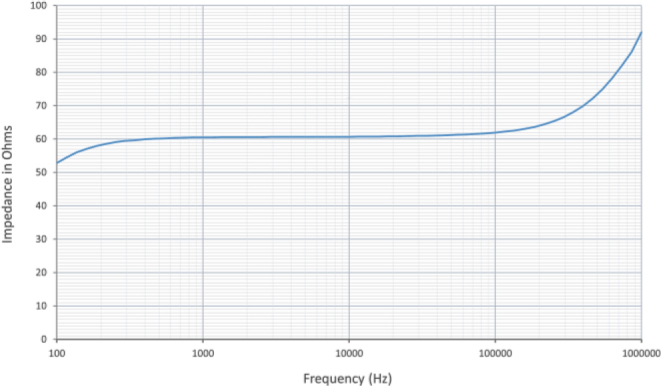
Variation of Impedance with Frequency of the control (1 ml Minimal Essential Medium, MEM).

The control used for this study is 1 ml of Minimal Essential Medium, MEM, described earlier. As shown in Figure 6, the impedance of cell culture medium can be considered linear for the frequencies described above. Based on the linear region shown in Figure 6, the measurement frequency for the experimental results was selected to be 1 kHz.

### Ethical approval

The conducted research is not related to either human or animal use.

## Results

Cell impedance was measured at definite intervals of L-929 mouse fibroblast cells established on the pin type electrode dishes. In a short time, 5 × 104 cells suspended in 1 ml MEM supplemented with Foetal Bovine Serum were transferred to electrode dishes and maintained in an incubator set at 37 ± 1 °C and 5% CO_2_.

Culture medium was replaced with fresh medium every alternate day. Cells were examined under phase contrast microscope (Leica DMRI, Germany) before each measurement.

The number of cells is minimum on day 1 and the number of cells is maximum on day 6 of the cell ceding, as seen both in microscope images and the impedance chart.

The impedance of the growing cell culture system was measured at 1, 2, 3, 5, 6 and 7 days, Figure 9. The L929 cells were able to adhere and form a monolayer. However, cells did not adhere at the interface of the plate and electrode due to the presence of adhesive. Further observations of cells around the glue showed that the adhesive used was not cytotoxic even though the cells fail to adhere on the adhesive. This study clearly demonstrated that the electrode configuration can effectively monitor the growth kinetics of cells. The observed impedance changes during the study correlated well with the microscope images.

**Fig.7:**
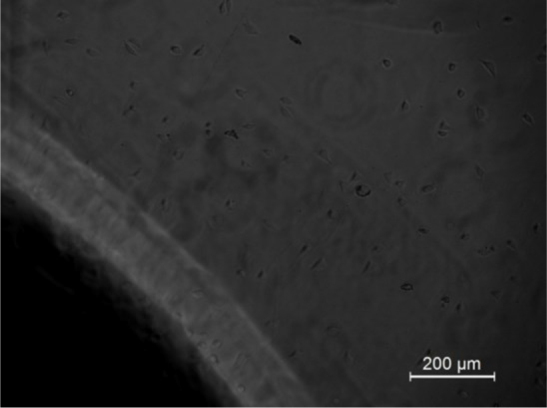
Microscope picture on day 1 of the cells seeding.

**Fig.8:**
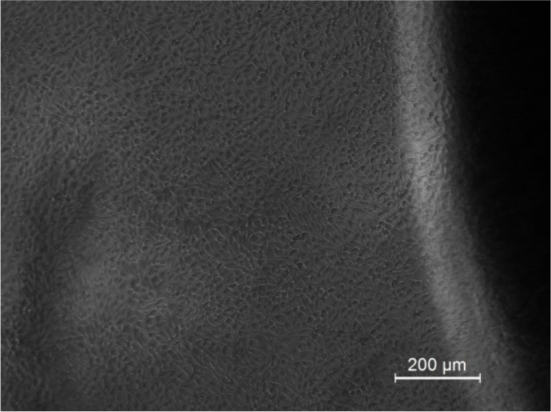
Microscope picture on day 6 of the cells seeding.

**Fig.9:**
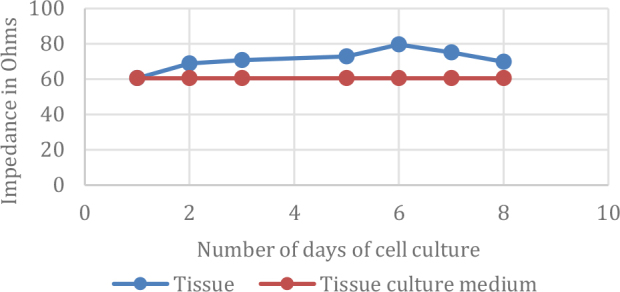
Variation of Impedance of cell culture against control (cell culture medium) measured at 1kHz.

As a number of cells proliferate and form a monolayer, the corresponding impedance changes can be clearly appreciated as depicted in Figures 7–9. The number of cells was maximum on day six and seven of the cell culture. As in any normal culture system, the cells were overgrown after the seventh day and started to deteriorate. The inception of cell death was marked by a change in the color of the tissue culture media (due to change of pH).

## Discussion

This study proposes a modified cell culture dish and a noninvasive method of analysis that can give an understanding of growth kinetics of cells at a macroscopic level. Historically, tetrapolar electrode configurations have been utilized to measure bioimpedance [9]. This study scientifically validates the concept of using tetrapolar electrode configuration impedance measurements to monitor cell growth in large cell culture plates, as opposed to using microelectrodes in multiwell plates. Measurement constraints at very low or high frequencies have been highlighted. Continuous monitoring has shown that impedance patterns are repetitive and are in unison with standard established technique of microscopy. The process of interfacing electrodes with a cell culture plate has been standardized.

Moreover, monitoring cell growth by impedance measurements has a unique advantage of being non-invasive i.e., without affecting the viability of cells for analysis, as compared to conventional microscopy techniques. The application of the proposed method can be extended to monitoring stem cells, cell adhesion and spreading on the surface of implants, monitoring wound healing as well as in toxicology studies. Nevertheless, this approach has limitations, such as providing solely the macroscopic depiction of cell growth kinetics, which can be addressed by calibration against a cell culture possessing known impedance and cell count.
